# British and Foreign Sailors' Society

**Published:** 1916-12-16

**Authors:** Margaret Lloyd George

**Affiliations:** 11 Downing Street, London, S.W.


### THE EDITOR'S LETTER-BOX.

### British and Foreign Sailors' Society.

*Letter from Mrs. Lloyd George.*

*To the Editor of* The Hospital.

Sir,—I venture to appeal to the sympathetic interest of your wide circle of readers in a cause which is very near my heart, and which, in a variety of ways, is voicing the Empire's gratitude to our sailors.

In addition to its lengthy programme of practical help, the British and Foreign Sailors' Society has been entrusted by the authorities with definite responsibility for the immediate welfare of aged mothers, widows, and orphans of the heroic seamen who have fallen whilst on active service; and, in spite of the many appeals of the present hour, I know of nothing more deserving of our generous support.

For, it must be remembered, in addition to all that can reasonably be expected from the State, there naturally remain considerable duties, which indeed can only be met through the generosity of large-hearted patriots.

It is estimated that at least £50,000 will be necessary for the great task the Sailors' Society has set itself, and knowing personally, as I do, its practical methods, its world-wide outlook, and its efficient administration, I ask your valued co-operation.

In view of the pressing need, it would *give me personal pleasure* to receive a generous donation from your readers. The smallest gifts will be more than welcome.—Believe me to be, very truly yours,

Margaret Lloyd George.

11 Downing Street, London, S.W.,
December 5, 1916.

### The Eradication of Venereal Disease.

*To the Editor of* The Hospital.

